# Psychometric properties of the Korean version of the Copenhagen Burnout Inventory in Korean homecare workers for older adults

**DOI:** 10.1371/journal.pone.0221323

**Published:** 2019-08-27

**Authors:** Gyeong-Suk Jeon, Sun-Ju You, Myo-Gyeong Kim, Yoo-Mi Kim, Sung-Il Cho

**Affiliations:** 1 Department of Nursing, Mokpo National University, Muan-gun, Jeollanam-do, Republic of Korea; 2 Institute of Health and Environment, Seoul National University, Seoul, Republic of Korea; 3 Department of Nursing, Seoul Women’s College of Nursing, Seoul, Republic of Korea; 4 Department of Health Policy and Management, Sangji University, Wonju-si, Kangwon-do, Republic of Korea; 5 Department of Public Health Sciences, Graduate School of Public Health, Seoul National University, Seoul, Republic of Korea; University of Malaya, MALAYSIA

## Abstract

**Background:**

Despite the increasing number of homecare workers, a reliable and valid tool with which to measure burnout among Korean homecare workers is still lacking. The aim of this study was to examine the reliability and construct validity of the Korean version of the Copenhagen Burnout Inventory (CBI-K).

**Methods:**

The study population consisted of 465 homecare workers. Data were collected in 2016 through a self-administered questionnaire including the three subscales of the CBI-K, the Center for Epidemiologic Studies Depression Scale (CESD-10), a measure of work–life conflict, and questions about respondents’ sociodemographic characteristics.

**Results:**

The confirmatory factor analyses results showed that the model fit indices of the refined three-factor model, in which the PB, WRB, and CRB subscales each contained six items, were acceptable (CFI = 0.924, SRMR = 0.049, RMSEA = 0.091). Furthermore, based on the results for construct reliability, discriminant validity of the refined three-factor model and job characteristics of homecare workers, we proposed that an abbreviated two-factor scale using the PB and CRB subscales could be used, with appropriate model fit indices (CFI = 0.950, SRMR = 0.047, RMSEA = 0.084). Each of the PB, WRB, and CRB subscales of CBI-K were associated with depressive symptoms even after controlling for covariates.

**Conclusions:**

The CBI-K has adequate reliability and validity for use with homecare workers. To increase its practicality, we suggest a refined form comprising only PB and CRB subscales can be used rather than a three-factor model.

## Introduction

Care workers are among the groups at greatest risk for the development of burnout syndrome. The service relationships that they develop with recipients require an ongoing and intense level of personal, emotional contact [1]. In particular, homecare workers working with older adults who are unable to independently perform the activities of daily living due to senile dementia or stroke perform almost all of these activities for them, including the minor tasks that have to be performed on a daily basis [2]. Moreover, those employed in homecare may be more susceptible to symptoms of caregiver burnout because they often work alone. Sole employment in private homes often exposes care workers to various dangers, including abusive behavior from the client and the client’s family, overexertion, unhygienic conditions, and so on [3]; it also limits opportunities for direct support from supervisors and coworkers [4]. These work environments can lead to stress and emotional as well as physical exhaustion [3].

Most industrialized countries are facing the aging of their populations. According to projections, by 2050, 30% of the population in Chile, China, Iran, Korea, the Russian Federation, Thailand, and Vietnam, and in many Organization for Economic Cooperation and Development (OECD) countries including those in Europe and North America, will be 60 years old or older [5]. The rapid pace at which the population of many countries is aging has led to concerns about the effectiveness and sustainability of extant systems for the long-term care of aging populations. Indeed, in many high-income countries, long-term care services are shifting from institutional care to home- and community-based models, which are more cost-effectiveness and preferred by older adults [6].

The Korean government has provided long-term care insurance (LTCI) for elderly individuals since 2008, and it initiated the social welfare voucher system in accordance with the Social Security Framework Act in 2007. According to LTCI statistics, 7.0% of older adults received LTCI in 2015; this was 1.67 times more than the 4.2% in 2008 (National Health Insurance Service, 2016). About 68.4% of LTCI recipients received care at home in 2016 [7]. The number of long-term care (LTC) workers also increased by more than 55% from 2005 to 2015, which makes Korea the OECD country with the second highest proportion (after Israel) of such workers [8]. In addition, more than 69% of LTC workers worked at private homes in 2016 [9].

Although the number of home-based LTCI recipients and care workers in Korea is increasing, the work environment of such workers is poor, and 95% of the social services workers in Korea are women [8]. These irregular part-time workers have low wages, low educational levels, and few skills [8, 10]. Compared to workers in other occupations, their employment stability and level of social protection are also relatively low [11]. Their lack of knowledge about how to provide care for recipients, the need to form interpersonal relationships with their clients, and their heavy workloads are the main contributors to the stress and burnout of care workers [12–15]. Despite the fact that the burnout of homecare workers may negatively affect both the health outcomes of care recipients and the psycho-social well-being of the workers themselves [16, 17], we still do not have a reliable and valid tool with which to measure burnout among Korean homecare workers.

According to Freudenberger [18] and Maslach [19], burnout is a work-related syndrome consisting of the following three dimensions: emotional exhaustion, depersonalization or cynicism, and reduced professional efficacy. Maslach and colleagues developed the Maslach Burnout Inventory (MBI) for measuring these three dimensions of burnout among professionals in the human service sectors [20] and then introduced a modified version of the MBI designed for all employment sectors [21]. The MBI is the most widely used standardized tool for research in this field and has been translated into and validated in many languages [22, 23]. However, despite its authoritative status and its monopoly in this field, it also has several limitations, such as the lack of a clear conceptual foundation, the lack of in-depth qualitative data on its use, and the difficulties involved in its administration [24–26]. In addition, according to several researchers, the exhaustion dimension considers only the emotional and not the physical and cognitive aspects of this phenomenon [27, 28]. These critiques reflect the MBI’s focus on measuring outcomes, which may constitute mixtures of heterogeneous processes. This feature may limit its validity in certain contexts.

Kristensen et al. [29] examined the etiology of burnout and identified exhaustion as the central aspect of burnout syndrome. They developed the Copenhagen Burnout Inventory (CBI) based on the distinction between physical and psychological exhaustion. This attention to causal relationships is a strength of studies of particular occupational groups as well as of efforts to develop intervention strategies. The CBI focuses on the core concept of burnout, including fatigue and exhaustion, and distinguishes among three different types of burnout: personal or generic burnout, which addresses the extent of physical and psychological exhaustion regardless of occupational status; work-related burnout, which addresses the degree of physical and psychological exhaustion that an individual attributes to work; and client-related burnout, which addresses the degree of physical and psychological exhaustion that an individual attributes to working with clients [29, 30]. Several recent studies that have validated the CBI in the human services [29, 31, 32] and other industrial [24] sectors have provided evidence of the strength of the CBI as a straightforward measure of burnout that, unlike the traditional measurement tool (the MBI), can also elucidate causal relationships (i.e., coping strategies).

Among the types of service work that are associated with vulnerability to burnout, homecare has been growing rapidly over the past decade, and this trend is expected to continue due to the aging of the global population. However, although the original study by Kristensen et al. included a homecare group [29], the application of CBI in this occupational group has been limited. As the working conditions of homecare workers differ from those of institution-based employees, further international validation studies with this population are needed. Korea is well known for its rapidly aging population and increase in homecare workers. An investigation of the applicability of the Korean version of the CBI (K-CBI) to care workers in Korea will enhance the usefulness of this tool. Therefore, in 2016, we performed confirmatory factor analyses (CFAs) of national representative data from care workers working with elderly individuals to examine the dimensionality of the K-CBI, to test the fit of the proposed dimensionality of the CBI measure and provide psychometric validation.

## Materials and methods

### Sample characteristics

A nationwide sample of care workers was selected via a two-stage stratified probability sampling design. In the first stage, investigators selected 100 care service centers including both private and public, which was about 5% of the 2,040 care service centers across the country registered with the Korea Social Security Information Service in 2015. This sample was stratified by 16 regions comprising seven metropolitan areas and nine provincial areas, as well as the size of the care service center. In the second stage, 500 care-workers were selected via proportional allocation based on the size of the sampling-unit institutions. A total of 471 care workers completed a self-administered questionnaire and provided informed consent, a response rate of 94.2%. During the survey, trained interviewers were available to assist anyone who might need any help. We excluded participants missing data for CBI and CESD-10 (n = 7), for a final sample of 464 homecare workers of private (32.3%) and public/non-profit (67.7%) homecare centers. All subjects gave their written informed consent for inclusion before participating in the study. The Institutional Review Board of M University in Korea provided approval for this study (project identification code 20160920-SB-008-01). Data were collected between July 2016 and October 2016.

### Assessment and measurements

#### Copenhagen Burnout Inventory (CBI)

We developed a Korean version of the CBI, which was originally developed by Kristensen et al. [29]. The CBI addresses three sub-dimensions: personal burnout (PB), work-related burnout (WRB), and client-related burnout (CRB). The three parts of the questionnaire were designed to be applied in different domains. The six items on the PB subscale measure feelings of physical, emotional, and mental fatigue and exhaustion. The WRB subscale contains seven items assessing the symptoms that respondents’ attribute to work. The six items on the CRB subscale describe feelings of physical and psychological fatigue and exhaustion that respondents attribute to their work with clients (i.e., patients). All items are scored on a five-point Likert scale; always/to a very high degree = 100, often/to a high degree = 75, sometimes/somewhat = 50, seldom/to a low degree = 25, and never /almost never/to a very low degree = 0. Item 4 of the WRB subscale (energy for others) was reverse scored. The score for each subscale is the average of item scores within the subscale. The range of scores on each subscale is 0–100. The average scores for each subscale among home helpers in the PUMA baseline study were PB 32.6, WRB 26.4, and CRB 26.2 [29].

We performed a translation and a back translation from the original English version of the CBI into Korean for this study. The translation of the CBI questionnaire into Korean was performed directly from the English version by two bilingual professionals whose first language was Korean and who had expertise in the field of job-related stress as well as worker health. The two bilingual professional interpreters independently back-translated the Korean version to English. An expert committee then compared the accuracy (the consistency of wording and information) of the back-translated English version to that of the original version to resolve discrepancies. Next, we performed a preliminary test of the final translated questionnaire with a sample drawn from the target population. Twelve Korean care workers completed the translated questionnaire and then were interviewed by two investigators (GS and SJ). All respondents reportedly understood the content of the items, and we concluded that the translated questionnaire was acceptable.

#### Depressive symptoms

We used depressive symptoms for evaluating the association between CBI-K and depression. Depressive symptoms were measured using the Korean version of the 10-item short-form Center for Epidemiological Studies Depression (CESD-10) scale, a brief screening instrument that assesses depressive symptoms experienced during the most recent week, treating it as a dependent variable. It consists of eight items addressing negative affect (loss of interest, trouble concentrating, feeling depressed, feeling tired or low in energy, feeling afraid, trouble falling asleep, feeling alone, and finding it hard to get going) and two items addressing positive affect (feel pretty good and generally satisfied). Each item is rated on a four-point scale: 0 = very rarely or less than once per day; 1 = sometimes or 1–2 days during the past week; 2 = often or 3–4 days during the past week; and 3 = almost always or 5–7 days during the past week. Scores on two positively phrased questions, items 5 and 8, were reversed. The scores on the 10 items were summed, resulting in total scores ranging from 0 to 30. Higher scores indicated more depressive symptoms. The alpha coefficient for the CESD-10 in this study was 0.73, which is lower than the 0.80 value obtained in previous reliability studies [33].

#### Covariates

Age (33–72 years), marital status (married vs. others), educational attainment (college or more, high school, middle school, elementary school or less), subjective economic status (high, middle, low), and work hours per week (4–104 hours) were included as covariates.

### Statistical analyses

Confirmatory factor analyses (CFA) of the K-CBI for care workers were performed to verify that the structure proposed by Kristensen et al. [29] had an adequate fit for this study’s sample. To carry out CFA, we first checked the normality of the data distribution of the CBI subscales, CESD-10, age, and working hours per week using skewness and kurtosis measures. All skewness and kurtosis values were within the range between −1 and 1, comparable to the multivariate normality of the CBI subscales. We employed the simple CFA model structure using the maximum likelihood estimation method. We also adjusted the model as follows. First, items with λ values <0.5 were dropped. Second, we allowed correlated error terms for the same factor based on modification indices estimated by Lagrange multipliers (LMs). The motive and justification for allowing correlated error terms were based on a theoretical rationale [34]. To compare the three-factor model with alternative models, we employed one-factor and two-factor models. In the two-factor model, the personal and work domains were specified to load onto the first factor, and the client domain.

To assess goodness of fit, we used comparative fit index (CFI), standardized root mean square residual (SRMR), and root mean square error of approximation (RMSEA). The model was considered an acceptable fit when the CFI, SRMR, and the RMSEA values were >0.95, <0.08, and <0.1 respectively [35, 36].

After evaluating the model fit, to verify the reliability of the measurement model in measuring the intended latent construct, we calculated composite reliability (CR); values of ≥0.7, were considered adequate [37]. To verify the discriminant validity that the items included in one factor were not correlated with another factor, we assessed the correlations between exogenous constructs (<0.85). We finally explored the association of the K-CBI subscales with measures of depressive symptoms using multiple regressions on depressive symptoms (CESD-10 total score). All regression models were adjusted for covariates (age, marital status, education, subjective economic status, and hours worked per week). We found no significant collinearity between any of the covariates in all regression analyses. The CFA was performed with AMOS 18.0, and other statistical tests were conducted using the Statistical Package for the Social Sciences software v. 22.0 for Windows (IBM Corp., Armonk, NY, USA).

## Results

Table 1 presents data on the sociodemographic characteristics, work situation, and health status of the study population. The average age of the participants was 56.76 years (range, 33–72). Most participants (81.0%) were married, and only 10.5% had graduated from college or attended graduate school; 75.9% rated their economic status as middle class. The average number of hours worked per week was 30.88 (range, 4–104), and 30.8% of homecare workers reported work–life conflict. The mean CESD-10 (SD) score was 7.80 (3.82), and 32.1% of respondents had scores greater than 10. The mean scores of personal, work-related, and client-related burnout were 38.59 (SD = 18.52), 33.94 (SD = 17.81), and 34.88 (SD = 18.36), respectively. Using a score of 50 or higher as a cutoff for a high degree of burnout, 30.4%, 23.3%, and 26.9% of participants reported a high level of burnout in the personal, work, and client-related domains, respectively.

**Table 1:** Sociodemographic characteristics, depressive symptoms, and burnout among Korean homecare workers working with community-dwelling elderly individuals (N = 464).

	Range	N or Mean	% or SD
Age (years)	33–72	56.76	6.68
≤54		163	35.1
55–64		240	51.7
≥65		61	13.1
Marital status			
Married		376	81.0
Widowed/other		88	19.0
Education			
College or more		49	10.5
High school		237	51.1
Middle school		127	27.4
Elementary school or less		51	11.0
Subjective economic status			
High		17	3.7
Middle		352	75.9
Low		95	20.5
Hours worked per week	4–104	30.88	21.84
Work–life conflict		143	30.8
Depressive symptoms scores^a^	0–18	7.80	3.82
CESD-10 scores ≥10		149	32.1
Personal burnout (six items)^b^	0–100	38.59	18.52
Scores ≥50		141	30.4
Work-related burnout (seven items)^b^	0–100	33.94	17.81
Scores ≥50		108	23.3
Client-related burnout (six items) ^b^	0–100	34.88	18.36
Scores ≥50		125	26.9

*Notes*: SD = Standard deviation.

^a^The 10-item short-form Center for Epidemiological Studies Depression scale.

^b^The original Copenhagen Burnout Inventory—Korean version

**Fig 1:**
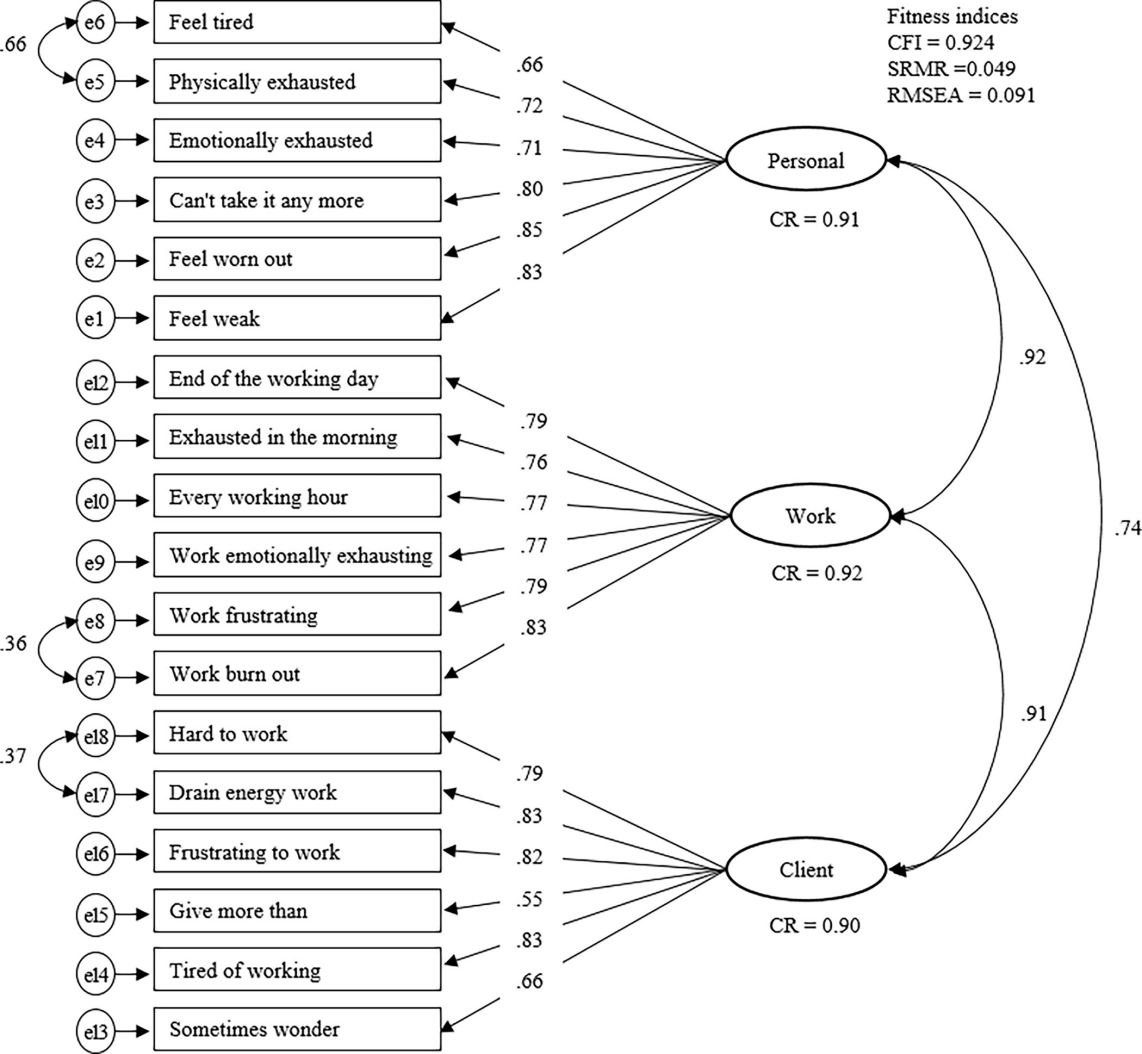
Results of the refined K-CBI model for Korean care workers. *Notes*: CFI: comparative fit index; SRMR: standardized root mean square residual; RMSEA: root mean square error of approximation; CR = composite reliability.

Table 2 displays indices of goodness of fit for the CFA results for the CBI-K. The original three-factor model exhibited inadequate goodness of fit for the sample used in this study (CFI = 0.866; SRMR = 0.056; RMSEA = 0.112). The two alternative models (one and two factor) displayed lower CFI and higher RMSEA values. In order to refine for better fitting models, item 4 on the WRB subscale, which had a low factorial weight (λ = 0.16), was removed. In addition, based on the modification indices calculated by LMs, we added error covariances between three adjoined pairs of items, items 1 and 2 on the PB subscale (MI = 202.946), items 6 and 7 on the WRB subscale (MI = 48.33), and items 1 and 2 on the CRB subscale (M = 32.784). The refined three-factor model was composed of the PB, WRB, and CRB subscales, with each having six items due to our removing one item and allowing three covariance. It exhibited acceptable goodness of fit for the sample used in this study (CFI = 0.924; SRMR = 0.049; RMSEA = 0.091) (Table 2). Finally, we tested for the refined two-factor model with PB and CRB excluding the WRB scale in the last step of CFA, considering that latent construct of work burnout showed too high correlation (≥0.85) with other two factors of PB and CRB (Fig 1). It exhibited acceptable goodness of fit (CFI = 0.950; SRMR = 0.047; RMSEA = 0.084) (Table 2).

**Table 2:** Goodness-of-fit indices of the factor models of the Korean version of the Copenhagen Burnout Inventory among Korean homecare workers working with elderly community-dwelling individuals (N = 464).

Step	Model description	Number of items	CFI	SRMR	RMSEA (90% CI)
1	Original three factor	19 = PB(6), WRB(7), CRB(6)	0.866	0.056	0.112 (0.106–0.119)
2	One factor	19 = PB(6)+WRB(7)+CRB(6)	0.790	0.070	0.139 (0.133–0.146)
3	Two-factor	19 = PB(6)+WRB(7), CRB(6)	0.838	0.064	0.123 (0.116–0.129)
4	Refined three-factor	18 = PB(6), WRB(6), CRB(6)	0.924	0.049	0.091 (0.083–0.096)
5	Refined two-factor	12 = PB(6), CRB(6)	0.950	0.047	0.084 (0.077–0.091)

*Note*. CFI: comparative fit index; SRMR: standardized root mean square residual; RMSEA (90% CI): root mean square error of approximation (90% confidence interval)

The values shown in Fig 1 are the standardized estimates of the covariance between the factors and the factor weights. All 18 items in the refined model of CBI-K had factorial weights (λ) ≥0.5. We found composite reliability of PB (CR = 0.91), WRB (CR = 0.92), and CRB (CR = 0.90) were exceed 0.7. Correlation between PB and CRB factors subscale were 0.74. Meanwhile, the correlation of WRB subscale with PB and CRB were greater than 0.85.

Table 3 presents the results of regression analyses between the refined CBI-K and the CESD-10. The refined CBI-K was associated with depressive symptoms, which indicates adequate concurrent validity of the refined CBI-K. The multiple regression, which were adjusted for covariates (age, marital status, education, subjective economic status, and hours worked per week), showed that PB (β = 0.389), WRB (β = 0.399), and CRB (β = 0.341) in the refined CBI-K were significantly associated with depressive symptoms. Dropping the WRB subscale from the regression model did not substantially change the results.

**Table 3:** The association between the revised Korean version of the Copenhagen Burnout Inventory and depressive symptoms among Korean care workers working with elderly community-dwelling individuals (N = 464).

	*β*	Adjusted *R*^*2*^
Personal burnout (six items)	0.389*	0.159
Work burnout (six items)	0.399*	0.165
Client burnout (six items)	0.341*	0.123

Notes

*P < 0.01

^a^regression analysis was adjusted for age, marital status, education, subjective economic status, and hours worked per week.

## Discussion

This is the first study to evaluate the psychometric properties of the CBI among homecare workers in Korea. We found the CBI-K burnout measures of the refined three-factor and two-factor models showed construct reliability, and they were also associated with depressive symptoms in the expected direction. However, only the refined two-factor model consisting PB and CRB secured good discriminant validity.

Although the revised three-factor model of the CBI-K exhibited an acceptable fit to the data, the current study elucidates several important aspects of the CBI-K as applied to homecare workers. First, item 4 of the WRB subscale (energy for others) was only weakly correlated with the other items and showed a low loading value (λ = 0.19) in the factor analysis. As this was the sole reverse-scored item on the CBI scale, participants may have responded to the item in a set manner without attending carefully to its content [32]. Therefore, we dropped the item for the revised three-factor model.

Second, in the revised three-factor model, we allowed three correlated error terms based on the modification indices calculated by LMs as well as theoretical justification. Fong et al. [32] also supported the adequacy of the revised three-factor CBI model, which added error covariance between three pairs of items, for a sample of human service workers working in a mental rehabilitation institution. The covariance of two adjoining items may be caused by their similar wording [32] and may be the result of similarity in respondents’ sensitivity to a specific condition. For example, the physical health status of respondents could contribute to both items 1 and 2 on the PB subscale. Although the modified model provided significant improvement in model fit, it should be noted that this could come from the post hoc nature of the procedure [34] or from chance.

Third, we found discriminant validity only between the PB and CRB subscales, but not between WRB and PB or WRB and CRB. Our finding is in line with previous studies showing a high correlation between personal and work burnout [24, 29, 32]. Low discriminant validity may arise from respondent or job characteristics. The respondents may not have perceived distinctions among the three CBI dimensions. However, the majorities of the participants in our study were younger than age 65 (86.7%) and had educational attainment of high school or more (61.7%). Therefore, the major influence on the discriminant validity may be the characteristics of homecare work. Our study subjects were homecare workers who worked an average of 30.88 hours per week at clients’ homes, thus it is not surprising that it was difficult to separate their experiences at work from those with clients. The main reason for the overlap between the WRB and CRB subscales is likely that our study subjects provided direct services. For example, their work was focused on meeting clients’ needs, and their places of work were clients’ own homes rather than agency offices or facilities. In general, care workers working with elderly individuals visit their agency only once per month. Indeed, care services, which are based on the interpersonal relationships between the service providers and the clients, can be understood as a type of emotional labor involving the assessment and satisfaction of the needs and desires of clients [38]. In this context, WRB is virtually synonymous with CRB, as “work” is precisely work with clients. As a result, it is unclear whether WRB is a necessary measure of work burnout for homecare workers. Indeed, the revised three-factor CBI-K subscales seemed to measure very similar phenomena in our sample population of homecare workers. Therefore, although the18-item CBI-K based on the three-factor model can be used for the purpose of comparison with other job categories, we suggest that the 12-item CBI-K based on a two-factor model using only the PB and CRB subscales would be more suitable for homecare workers, in consideration of the construct reliability and discriminant validity results, job characteristics of homecare workers, and the better model fit of the two-factor model (six PB items and six CRB items) compared with those of the three-factor model (six PB, six WRB, and six CRB items).

Regarding the CBI score, Korean care workers who work with community-dwelling older adults scored higher on the PB subscale (M = 38.58, SD = 18.50) than did homecare workers in a province in Denmark (M = 32.6), and lower than homecare workers in the capital of Denmark (M = 43.1) [29]. Scores on the CRB subscale (M = 34.89, SD = 18.35) fell between those of homecare workers in the capital (M = 35.9) and those in a province (M = 26.2) in Denmark [29]. There are several possible reasons for the differences, including working conditions, socioeconomic status and wages, access to stress-management programs, and cultural context, although the authors of the Danish study [29] did not provide explanations for the differences between home helpers in the capital and those in the province.

In a globalized society in which most physically laborious jobs are now performed by machines, interpersonal caregiving work may become more valuable, particularly in the context of the increasing older population. As the work environment evolves in sync with social changes, we need to continue to assess our measures. This study imply that the CBI-K is currently useful for identifying homecare workers at risk and employing preventive strategies and measures, although it may need to be adapted to suit the changing work environment. Our findings could also use as a validation reference for other homecare workers such as Korean home-helpers for disabled persons.

This study has some limitations. The results presented should be considered in light of the limitations associated with our use of a cross-sectional study and a sample of 464 individuals. That is, our findings only apply to burnout among homecare workers. Further assessment of the validity and reliability of the Korean version of the CBI for other care worker groups could enhance its validity. The RMSEA values of the refined three-factor (0.091) and two-factor model (0.084) which indicate "mediocre" fit could be a limitation. However, other indices (CFI and SRMR) which are in a range suggesting "good" model fit can attenuate our concern [39]. Finally, given the significant impact of sociodemographic factors on homecare workers’ burnout future research for exploring these predictive variables could be suggested.

Despite these limitations, it is noteworthy that, notwithstanding the fact that versions of the CBI are already available in a variety of languages (e.g., English, Japanese, Mandarin, Catalan, Swedish, Finnish, French, Danish, Portuguese) and the fact that it has been tested on several different groups [24, 27, 29, 40], this is the first study that applied the Korean version of the instrument to a sample of care workers working with elderly individuals. Both the consistency of our findings with those reported in the literature as well as our actual results confirm the reliability and validity of the CBI and support our recommendation for its use to screen for burnout syndrome in homecare workers in Korea.

## Conclusions

Burnout syndrome should be a focus of concern for service agencies as well as health authorities because of its impact of the physical and psychological well-being of workers. From this perspective, it is crucial that measurement and diagnostic instruments that are adequately calibrated to the target population are available. Our study contributes to the evaluation of the psychometric properties of the CBI for use with a population of elderly care workers in Korea. The results, showing the refined CBI-K has adequate reliability and validity, indicate that the Korean version of the inventory for homecare workers is an adequate tool for assessing the occurrence of burnout syndrome and thus is a useful tool for agencies and health authorities. In addition, the refined short version based on the two-factor model could be practically more proper to use for homecare workers.

## Supporting information

S1 Fig Questionnaire of Korean version of Cophenhagen Burnout Inventory(CBI-K). (GIF)

S1 File Minimum data set for CBI-K are available from Figshare (https://figshare.com/s/960e125ab939b19d8111). 10.6084/m9.figshare.7212485 (The DOI becomes active when the item is published).
(SAV)

## References

[1] Maslach C, Leiter MP. Understanding the burnout experience: recent research and its implications for psychiatry. World Psychiatry. 2016;15(2):103–111. 10.1002/wps.20311 27265691 PMC4911781

[2] Chon KN, Park OI, Moon H. The effect of care giver's knowledge and attitude toward the elderly on job stress. Korean J. Community Living Sci. 2010;21(1):19–32. [Korean]

[3] National Institute for Occupational Safety and Health. NOISH Hazard review: Occupational hazards in home healthcare. Available from: https://www.cdc.gov/niosh/docs/2010-125/default.html Cited 4 March 2018.

[4] Hurrell J, Murphy L. Psychological job stress in Environmental and occupational medicine 2nd ed.; Rom, W.; Little and Brown: Boston, MA, USA; 1992 pp. 675–674.

[5] World Health Organization. World report on ageing and health 2015 Available from: http://www.who.int/ageing/events/world-report-2015-launch/en/ Cited 4 March 2018.

[6] World Health Organization. Home Care across Europe: Current structure and future challenges. Available from: http://www.euro.who.int/__data/assets/pdf_file/0008/181799/e96757.pdf. Cited 4 March 2018.

[7] National Health Insurance Service. 2015 Statistics of Long Term Care Insurance. Available from: http://www.nhis.or.kr/menu/retriveMenuSet.xx?menuId=F332a. Cited 28 Feb 2018.

[8] OECD. Health at a Glance 2017 OECD Indicators. Available from: http://www.oecd-library.org/social-issues-migration-health/health-at-a-glance-2017_health_glance-2017-en. Cited 28 Feb 2018.

[9] National Health Insurance Service. 2016 Statistics of Long Term Care Insurance. Available from: http://www.nhis.or.kr/menu/retriveMenuSet.xx?menuId=F332a. Cited 28 Feb 2018.

[10] Kim BH. A study on the job satisfaction for Employees Working with the social service voucher system Master's thesis, Chosun University: Gwangju, Korea, 2010. [Korean]

[11] Yoon JY. The status and tasks of working conditions for social care service in Monthly Labor Review; Korea Labor Institute: Sejong, Korea; 2012 pp. 71–83. [Korean]

[12] Kennedy BR. Stress and burnout of nursing staff working with geriatric clients in long-term care. J Nurs Scholarsh. 2005;37(4):381–382. 16396413 10.1111/j.1547-5069.2005.00065.x

[13] Choi HK. A study on direct care work as emotional labor in nursing facilities. Korean Journal of Social Welfare Research. 2011;29:113–138.

[14] Chon YH. A study on relationships between the elderly with dementia and care workers under the long-term care insurance for the elderly in Korea. Studies on Life and Culture. 2017;43:129–171.

[15] Jeon GS, You SJ, Kim MG, Kim YM. Correlates of depressive symptoms and stress among Korean women care-workers for older adults dwelling in community. Korean J Occup Health Nurs. 2017;26(1):10–18.

[16] Natan MB, Lowenstein A, Eisikovits Z. Psycho-social factors affecting elders’ maltreatment in long-term care facilities. Int Nurs Rev. 2010;57(1):113–20. 10.1111/j.1466-7657.2009.00771.x 20487483

[17] Poghosyan L, Clarke SP, Finlayson M, Aiken LH. Nurse burnout and quality of care: Cross-national investigation in six countries. Res Nurs Health. 2010;33:288–298. 10.1002/nur.20383 20645421 PMC2908908

[18] Freudenberger HJ. Staff burn-out. Journal of Social Issues. 1974;30:159–65.

[19] Maslach C. Burned-out. Human Behavior. 1976;5:16–22.

[20] Maslach C, Jackson S. Maslach Burnout Inventory Manual, 3rd ed.; Consulting Psychologists Press: Palo Alto, CA, USA; 1986.

[21] Maslach C, Jackson S, Leiter MP. Maslach Burnout Inventory Manual, 3rd ed.; Consulting Psychologists Press: Palo Alto, CA, USA; 1996.

[22] Maslach C, Leiter MP, Schaufeli WB. Measuring burnout In The Oxford handbook of organizational well-being, 1st ed,; Cooper C.L., Cartwright S.; Oxford University Press: New York, NJ, USA; 2009 pp. 86–108, ISBN-10: 0199211914

[23] Schaufeli W, Buunk B. Burnout: An overview of 25 years of research and theorizing In The handbook of work and health psychology, Schabracq M.J., Winnubst J.A.M, Cooper C.L.; John Wiley & Sons: Chichester, West Sussex, UK; 2003. pp. 383–429, ISBN-10: 0471892769

[24] Yeh WY, Cheng Y, Chen CJ, Hu PY, Kristensen TS. Psychometric Properties of the Chinese Version of Copenhagen Burnout Inventory Among Employees in Two Companies in Taiwan. Int J Behav Med. 2007;14(3):126–133. 18062055 10.1007/BF03000183

[25] Korczak D, Huber B, Kister C. Differential diagnostic of the burnout syndrome. GMS Health Technol Assess. 2010;6:Doc 09. 10.3205/hta000087 21289882 PMC3010892

[26] Winwood PC, Winefield AH, Lushington K. The role of occupational stress in the maladaptive use of alcohol by dentists: A study of south Australian general dental practitioners. Aust Dent J. 2003;48(2):102–109. 14649399 10.1111/j.1834-7819.2003.tb00017.x

[27] Milfont TL, Denny S, Ameratunga S, Robinson E, Merry S. Burnout and wellbeing: Testing the Copenhagen Burnout Inventory in New Zealand teachers. Social Indicators Research. 2008;89(1):169–177. 10.1007/s11205-007-9229-9

[28] Demerouti E, Bakker AB, Nachreiner F, Schaufeli WB. The job demands-resources model of burnout. J Appl Psychol. 2001;86(3):499–512. 11419809

[29] Kristensen TS, Borritz M, Villadsen E, Christensen KB. The Copenhagen Burnout Inventory: A new tool for the assessment of burnout. Work and Stress. 2005;19(3):192–207. 10.1080/02678370500297720

[30] Borritz M, Rugulies R, Bjorner JB, Villadsen E, Mikkelsen OA, Kristensen TS. Burnout among employees in human service work: design and baseline findings of the PUMA study. Scand J Public Health. 2006;34(1):49–58. 10.1080/14034940510032275 16449044

[31] Molinero Ruiz E, Quintero H BG, Moncada Lluis S. Validation of the Spanish version of the Copenhagen Burnout Inventory Questionnaire. Rev Esp Salud Publica. 2013;87:165–179. 10.4321/S1135-57272013000200006 23775105

[32] Fong TC, Ho RT, Ng SM. Psychometric Properties of the Copenhagen Burnout Inventory-Chinese Version. J Psychol. 2014;148(3):255–66. 10.1080/00223980.2013.781498 24839726

[33] Irwin M, Artin KH, Oxman MN. Screening for depression in the older adults; criterion validity of the 10-item center for epidemiological studies depression scale (CES-D). Arch Intern Med. 1999;159:1701–1704. 10.1001/archinte.159.15.1701 10448771

[34] Hermida R. The problem of allowing correlated errors in structural equation modeling: concerns and considerations. Comput Models Soc Sci. 2015; 3(1):5–17.

[35] Shadfar S, Malekmohammad I. Application of Structural Equation Modeling (SEM) in restructuring state intervention strategies toward paddy production development. Int. Journal of Academic Research in Business & Social Sciences. 2013; 3(12):579–618.

[36] McDonald RP, Ho MH. Principles and practice in reporting structural equation analyses. Psychol Methods. 2002;7(1):64–82. 10.1037//1082-989x.7.1.64 11928891

[37] Fornell C, Larcker DF. Evaluating Structural Equation Models with unobservable variables and measurement error. J Mark Res. 1981;18(1):39–50.

[38] Ungerson C. Care, work and feeling. Sociol Rev. 2005;53:188–203. 10.1111/j.1467-954X.2005.00580.x

[39] Brown TA. Confirmatory factor analysis for applied research 2nd ed. New York: The Guilford press; 2006 pp. 86–87.

[40] Campos JA, Carlotto MS, Marôco J. Copenhagen Burnout Inventory—student version: adaptation and transcultural validation for Portugal and Brazil. Psicologia: Reflexão e Crítica 2013;26(1):87–97.

