# Supplementary figures and images for: Psychometric properties of the Korean version of the Copenhagen Burnout Inventory in Korean homecare workers for older adults
Source: PLoS One. 2019 Aug 27;14(8):e0221323. doi: 10.1371/journal.pone.0221323 (PMC6711598; doi:10.1371/journal.pone.0221323)

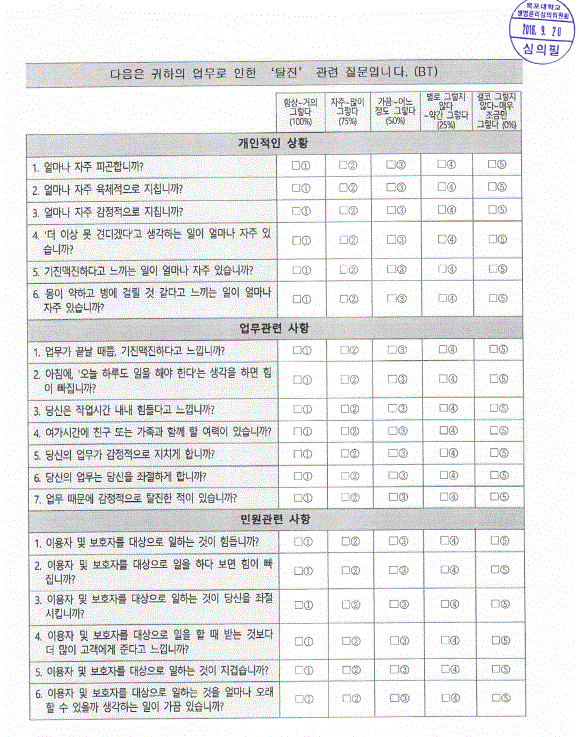

Supplement: S1 Fig — (GIF) [file pone.0221323.s001.GIF]
